# Multi-trajectories of lipid indices with incident cardiovascular disease, heart failure, and all-cause mortality: 23 years follow-up of two US cohort studies

**DOI:** 10.1186/s12967-021-02966-4

**Published:** 2021-07-03

**Authors:** Fatemeh Koohi, Davood Khalili, Mohammad Ali Mansournia, Farzad Hadaegh, Hamid Soori

**Affiliations:** 1 Department of Epidemiology, School of Public Health and Safety, Shahid Beheshti University of Medical Sciences, Tehran, Iran; 2 Department of Epidemiology and Biostatistics, Research Institute for Endocrine Sciences, Shahid Beheshti University of Medical Sciences, Tehran, Iran; 3 Prevention of Metabolic Disorders Research Center, Research Institute for Endocrine Sciences, Shahid Beheshti University of Medical Sciences, Tehran, Iran; 4 Department of Epidemiology and Biostatistics, School of Public Health, Tehran University of Medical Sciences, Tehran, Iran; 5 Safety Promotion and Injury Prevention Research Center, Shahid Beheshti University of Medical Sciences, Tehran, Iran

**Keywords:** Multi-trajectories, Lipids, HDL cholesterol, LDL cholesterol, Triglycerides, Cardiovascular disease, Heart failure

## Abstract

**Background:**

Understanding the distinct patterns (trajectories) of variation in blood lipid levels before diagnosing cardiovascular disease (CVD) might carry important implications for improving disease prevention or treatment.

**Methods:**

We investigated 14,373 participants (45.5% men) aged 45–84 from two large US prospective cohort studies with a median of 23 years follow-up. First, we jointly estimated developmental trajectories of lipid indices, including low-density lipoprotein cholesterol (LDL-C), high-density lipoprotein cholesterol (HDL-C), and triglyceride (TG) concentrations using group-based multi-trajectory modeling. Then, the association of identified multi-trajectories with incident CVD, heart failure, and all-cause mortality were examined using Cox proportional hazard model.

**Results:**

Seven distinct multi-trajectories were identified. The majority of participants (approximately 80%) exhibited decreasing LDL-C but rising TG levels and relatively stable HDL-C levels. Compared to the individuals with healthy and stable LDL-C, HDL-C, and TG levels, those in other groups were at significant risk of incident CVD after adjusting for other conventional risk factors. Individuals with the highest but decreasing LDL-C and borderline high and rising TG levels over time were at the highest risk than those in other groups with a 2.22-fold risk of CVD. Also, those with the highest and increased triglyceride levels over time, over optimal and decreasing LDL-C levels, and the lowest HDL-C profile had a nearly 1.84 times CVD risk. Even individuals in the multi-trajectory group with the highest HDL-C, optimal LDL-C, and optimal TG levels had a significant risk (HR, 1.45; 95% CI 1.02–2.08). Furthermore, only those with the highest HDL-C profile increased the risk of heart failure by 1.5-fold (95% CI 1.07–2.06).

**Conclusions:**

The trajectories and risk of CVD identified in this study demonstrated that despite a decline in LDL-C over time, a significant amount of residual risk for CVD remains. These findings suggest the impact of the increasing trend of TG on CVD risk and emphasize the importance of assessing the lipid levels at each visit and undertaking potential interventions that lower triglyceride concentrations to reduce the residual risk of CVD, even among those with the optimal LDL-C level.

**Supplementary Information:**

The online version contains supplementary material available at 10.1186/s12967-021-02966-4.

## Introduction

Cardiovascular diseases (CVDs) remain the leading cause of death and a significant cause of disability worldwide, accounting for 17.9 million deaths per year or 31% of all global deaths [1]. Unfavorable lipid indices, represented by increased serum concentrations of total cholesterol (TC), triglyceride (TG), low‐density lipoprotein cholesterol (LDL‐C), and decreased high-density lipoprotein cholesterol (HDL‐C), are conventionally considered to play an essential role in the development and progression of cardiovascular disease [2].

Some existing evidence suggests age-related changes in the TC, LDL-C, and TG so that they increase up to middle age and then decrease [3–6]. However, some epidemiological evidence also suggests that long-term exposure to even moderately raised cholesterol levels is associated with CVD in the future [7, 8]. Therefore, understanding the distinct patterns of variation in blood lipid levels before the diagnosis of CVD might carry important implications for improving disease prevention or treatment.

Most of the evidence on the trends in lipid indices comes from comparing observed average lipid levels in sequential cross-sectional surveys, and only a few of these studies have investigated trends in the lipid indices within the same population [3, 9–12]. However, most of these investigations did not consider the correlation between lipids and assessed the trajectory of lipid indices separately [3, 10–12]. Thus, evidence exists on lipid indices’ independent effect, but little is known about their combined impact on CVD risk. Moreover, identifying distinct longitudinal patterns of different lipids might help better understand the variation of lipid indices over time and facilitate targeted cardiovascular prevention programs [9].

Using pooled data from two US cohort studies with a median of 23 years follow-up and repeated measurements of lipid indices, we sought to (1) identify longitudinal multi-trajectories of LDL‐C, HDL‐C, and TG over the adult life course and (2) examine their associations with subsequent risks of incident CVD, heart failure, and all-cause mortality later in life. To capture the overlap between the developments of LDL‐C, HDL‐C, and TG, we estimated the joint developmental trajectories of these lipids. This approach allowed us to identify subgroups that share common patterns of change over time.

## Materials and methods

### Study population

The present study was based on data from two large, community-based, prospective cohort studies sponsored by the National Heart, Lung, and Blood Institute (NHLBI), the Atherosclerosis Risk in Communities (ARIC) study, and the Multi-Ethnic Study of Atherosclerosis (MESA) study. Details of the method and design of each study have been previously published [13, 14]. In brief, the ARIC Study is a prospective cohort study of 15,792 individuals 45 to 64 years of age recruited from 1987 to 1989 from 4 US communities [13]. The MESA Study recruited 6814 individuals 45 to 84 years of age free of clinical CVD at baseline during 2000–2002, from 6 US communities [14]. So far, both studies have five subsequent examination cycles (a sixth follow-up visit is currently underway), and all participants provided written informed consent. The studies’ website contains details of all the available data through a fully searchable data dictionary [15, 16]. The process of pooling data was carried out using the guidelines developed by Maelstrom Research for rigorous retrospective data harmonization [17].

**Fig. 1:**
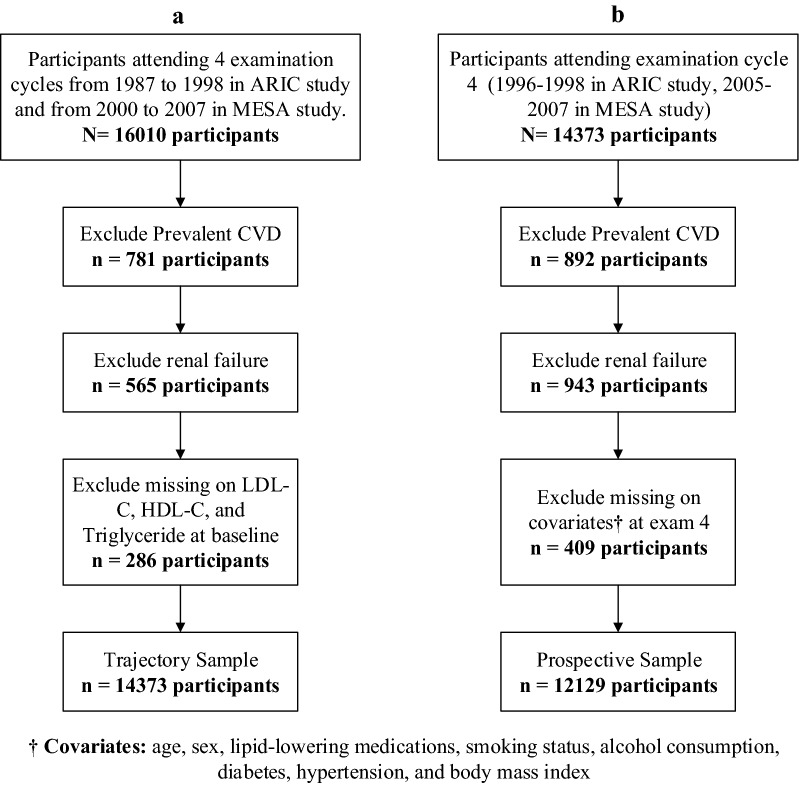
The flowchart of the participant selection process in the current study. Sample **a** for trajectory analysis; sample **b** for prospective (survival) analysis

The current study consists of two samples according to the analyses performed. The flowchart of the participant selection process is shown in Fig. 1. The first sample (sample A) was used to create the multi-trajectory groups for the lipid indices with the objective of a later link to CVD events and all-cause mortality. Since the trajectory approach requires at least three unique time points and becomes more precise with additional time points, this sample was restricted to participants attending all 4-examination cycles. Besides, participants with known CVD and renal failure and missing values for each lipid at baseline were excluded from this sample (Fig. 1, sample A). For incident CVD events from the second exam through the fourth exam, all data until the date of the first incident CVD have been included. Data from examination cycle five was not used for prospective analyses described as follows to ensure sufficient follow-up time for these analyses.

The second sample (sample B) was used in prospective (survival) analyses to link multi-trajectory groups of lipid indices (defined using sample A) with incident CVD, heart failure, and all-cause mortality later in life. This sample (sample B) included participants in sample A who attended the fourth examination cycle and did not have prevalent CVD, renal failure, and missing data on covariates on examination cycle 4 (Fig. 1, sample B). These participants were followed from examination cycle four until December 2014.

### Assessment of the lipid indices and covariates

In both studies, demographic characteristics and CVD risk factors were measured using standardized protocols and similar standard and validated methods at each examination cycle. In the current analysis, we used the repeated blood lipid measurements, including LDL-C, HDL-C, and triglycerides (TG).

At each examination cycle, blood samples were collected after a 12-h fast using a standardized venipuncture procedure. EDTA plasma samples were aliquoted on ice and stored at − 70 °C until analysis. Total cholesterol and triglycerides were measured using standard enzymatic processes (Roche Diagnostics). After precipitation of non-HDL-cholesterol with magnesium/dextran, the HDL-C level was measured using the cholesterol oxidase method (Roche Diagnostics). The concentration of LDL-C was calculated from the concentrations of total cholesterol, HDL-C, and triglyceride values < 400 mg/dL by the Friedewald formula. Arterial blood pressure was measured three times on the right arm and in a sitting position after a 5-min rest; the average value of the second and third measurements was used in the analysis. Information on smoking status, alcohol consumption, medications for lowering lipid levels, blood pressure, and diabetes mellitus was obtained from standard questionnaires and bringing in drugs by participants in each examination cycle.

### Outcomes

In this study, the primary outcomes of interest for the prospective analysis were incident cardiovascular disease (All-CVD defined as MI, Resuscitated Cardiac Arrest, CHD Death, Stroke, or Stroke Death), heart failure, and all-cause mortality. For these analyses, participants were followed from examination cycle 4 (1996–98 in the ARIC and 2005–2007 in the MESA study) through 2014. In both studies, events were ascertained and adjudicated using each cohort’s specific protocol. All events were ascertained by following each participant at intervals of 9–12 months using telephone calls. Then, a review committee through medical records and death certificates in both studies adjudicated them for end-point classification and assignment of incidence dates.

### Statistical analysis

Descriptive statistics, as mean (SD) for continuous variables or as frequencies (%) for categorical variables, were reported for the trajectory sample (sample A) using characteristics measured at the first examination cycle and for the prospective sample (sample B) using data at the fourth examination cycle.

We used the group-based multi-trajectory modeling (GBMTM) with age as the time scale to explore the jointly longitudinal changes of lipid indices. We implemented this technique using Proc Traj [18] in Stata software version 14 (STATA Corp., TX, US). Briefly, this model is a new application of group-based trajectory modeling (GBTM). GBMTM is a semiparametric mixture model, which allows the joint modeling of the trajectories of multiple outcomes. This model identifies latent clusters of individuals who follow similar patterns through multiple outcomes using a maximum likelihood method [18–20]. In the GBTM models, each individual is assumed to belong to only one group, where each group has a distinct trajectory. We applied a censored normal model [21] to identify distinct trajectories of lipid concentrations.

This study jointly estimated developmental trajectories of HDL-C, LDL-C, and TG, given that all these lipids completely determine total cholesterol. Values of TG were log-transformed because of its skewed distribution. Varied GBMT models were run before selecting the best model regarding the number of groups and trajectory shapes (e.g., constant, linear, quadratic, cubic) [20, 22]. First, to identify the optimal number of distinct groups to describe heterogeneity in the longitudinal development of lipid indices, various models using 3–9 distinct groups with fixed slope variance within groups were fitted. Then, quadratic slopes were added to the model allowing for curved developmental patterns. The improvement in model fit gained by adding additional groups or shape parameters was assessed based on the Bayesian information criteria (BICs) [22]. When comparing two models with different groups or trajectory shapes, the Bayes factor was also estimated by exp^(BIC1−BIC2)^, where BIC1 and BIC2 represent the BIC values for models 1 and 2, respectively, to assess significant change in BIC value. A tenfold difference in the Bayes factor is considered a significant difference [22]. A model with the least BICs and sufficient sample size in each multi-trajectory group (> 5% of the sample) was chosen as the best model. Finally, to ensure that our chosen model fits the data well, we assessed four model's fit diagnostic criteria as suggested by Nagin [21]: (1) an average posterior probability of assignment for each group j (AvePPj) equal to 0.7 or greater for all groups that are considered as good discrimination in classifying individuals into distinctive groups; (2) the odds of correct classification (OCCj) equal to 5 or higher for all groups; (3) reasonable similarity between the proportion of a sample assigned to a specific group and the group probabilities estimated from the model; and (4) narrow CIs of the estimated proportion.

Since the interaction term between sex and multi-trajectory groups was not statistically significant, and the overall pattern of trajectories was similar in men and women, we used a GBMTM including all participants without sex stratification. Using this algorithm, we identified seven distinct multi-trajectories for HDL-C, LDL-C, and TG. Demographic and health characteristics of the final lipid components multi-trajectory groupings were compared using Pearson’s chi-squared tests (categorical variables) or analysis of variance (ANOVA) (continuous variables).

Finally, we assessed the associations of multi-trajectory group membership (modeled as a categorical independent variable) and incidence of CVD, heart failure, and all-cause mortality on follow-up after exam four. To do so, we conducted separate Cox proportional hazards regression models for each outcome, adjusting for age, sex, race, lipid-lowering medications, smoking status, alcohol consumption, diabetes, hypertension, and BMI measured at exam four.

Finally, as a sensitivity analysis, we repeated survival analyses adjusting for the covariates mentioned above and total caloric intake and physical activity at baseline since we have data on both covariates just at the baseline exam. Collinearity between lipid indices and lifestyle factors, including physical activity, total caloric intake, smoking, and alcohol consumption, was tested using Pearson’s correlation and a variance inflation factor (VIF), which revealed no significant collinearity (VIF < 2 for all variables, Additional file 1: Table S1).

## Results

### Sample characteristics

The baseline characteristics of the participants are shown in Table 1. A total of 14,373 participants aged 45–84 were included in the trajectory sample to identify the developmental multi-trajectory patterns of LDL-C, HDL-C, and TG (up to 4 visits); 6534 (45.5%) were men, the mean (SD) age at baseline was 56.3 (8.1) years, and 9593 (67.7%) were white. The association between lipid indices’ multi-trajectory groups and incident CVD and all-cause mortality were examined among 12,129 participants; 5379 (44.4%) were men, the mean (SD) age at examination cycle 4 was 63.3 (7.3) years, and 8140 (67.1%) were white.

**Table 1:** Baseline characteristics of the participants

Characteristics	Trajectory sample (n = 14,373)	Prospective sample (n = 12,129)
Age, y	56.3 ± 8.1	63.3 ± 7.3
Sex		
Women, n (%)	7839 (54.5)	6750 (55.7)
Men, n (%)	6534 (45.5)	5379 (44.4)
Race		
White/Caucasian	9593 (66.7)	8140 (67.1)
African American/Black	3138 (22.0)	2605 (21.5)
Hispanic/Latino	1044 (7.3)	889 (7.3)
Asian/Chinese	571 (4.0)	495 (4.1)
Body mass index, kg/m^2^	27.6 (5.2)	28.4 (5.5)
Total cholesterol, mg/dL	206.7 (39.4)	197.3 (36.7)
LDL cholesterol, mg/dL	129.9 (36.8)	119.3 (33.1)
HDL cholesterol, mg/dL	52.2 (16.2)	51.7 (16.3)
Triglycerides, mg/dL	122.7 (63.6)	132.8 (77.4)
Systolic blood pressure, mmHg	120.9 (18.5)	125.1 (19.0)
Diastolic blood pressure, mmHg	72.5 (10.4)	70.6 (9.9)
Hypertension	4585 (32.0)	5271 (43.5)
Antihypertensive drug	3810 (26.5)	4586 (37.8)
Lipid-lowering drug use	2609 (18.2)	3799 (31.3)
Diabetes mellitus	1269 (8.8)	1730 (14.3)
Current smoking	2634 (18.4)	1569 (12.9)
Current drinking	8510 (59.5)	6114 (50.4)

Numbers represent mean (standard deviation) for continuous and frequency (%) for categorical variables

Characteristics for the trajectory sample were measured at the baseline (the first examination cycle)

Characteristics for the prospective sample were measured at the fourth examination cycle

*HDL* high-density lipoprotein, *LDL* low-density lipoprotein

### Characterization of multi-trajectories of lipid indices

Using the procedure and criteria mentioned above, we chose the 7-group multi-trajectory model from all investigated models. Table 2 indicates the estimates of the diagnostic criteria for judging the adequacy of the final model. Average posterior probabilities were high for all seven groups (range, 0.85–0.95) and the odds of correct classification were all well above 5. In all seven multi-trajectory groups, the average posterior probability (AvePP) was greater than 0.85, far greater than the recommended value of 0.7, indicating that the model assigned individuals to different multi-trajectory groups with little ambiguity. Further, the value for the OCC was greater than 20 for all seven groups, which is also greater than the recommendation of 5 as a general guideline for GBTM [22].

**Table 2:** Diagnostic criteria for judging the adequacy of the final model

Trajectory group	AvePP	OCC	P	π
Group 1	0.88	20.2	0.257	0.251
Group 2	0.89	35.7	0.188	0.187
Group 3	0.91	50.6	0.168	0.168
Group 4	0.85	50.5	0.094	0.098
Group 5	0.89	55.0	0.130	0.131
Group 6	0.95	321.9	0.060	0.060
Group 7	0.86	52.7	0.103	0.105

AvePP: average posterior probability; OCC: odds of correct classification; p: actual proportion of subjects assigned to each trajectory group using the maximum probability rule; π: the posterior probability of group membership estimated by the model

**Fig. 2:**
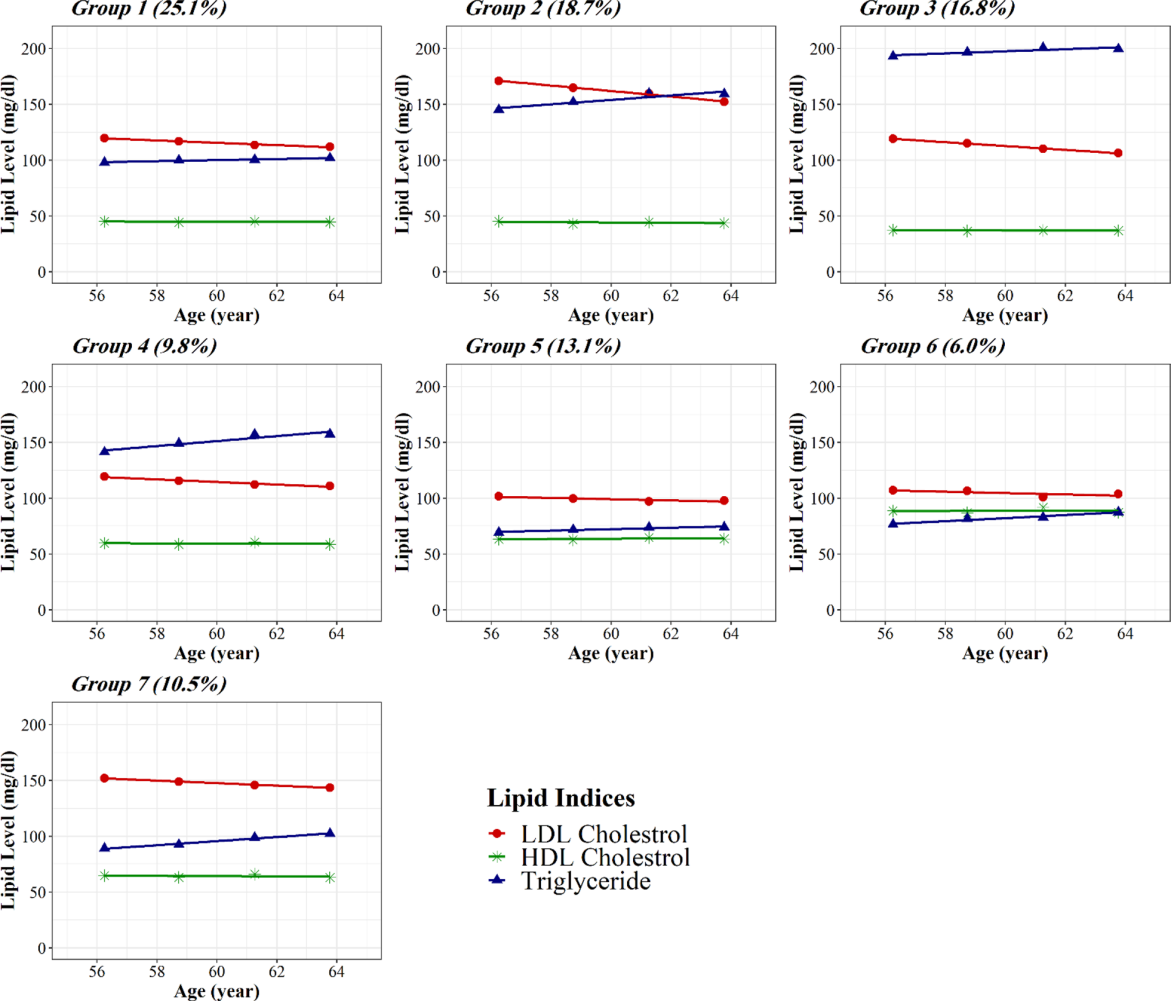
Multi-trajectory groups of LDL-C, HDL-C, and TG using group-based multi-trajectory modeling. Dots show group-specific mean observed levels while solid lines represent fitted trajectories. Lipids were modeled as a function of age, with lipid-lowering medication usage included as a time-varying covariate

Figure 2 shows the plot of the multi-trajectory groups of lipid indices and expected group percentages for each of the groups. The majority of participants (approximately 80%) exhibited decreasing LDL-C but rising TG levels and relatively stable HDL-C levels. Groups one to four and seven have worse values than the rest for most considered lipids, with groups two and three being uniformly worse and the other three groups (one, four, and seven) each having one or two for which values are high. Indeed, group two has worse but decreasing concentrations in LDL-C and borderline high but increasing in TG, representing 18.7% of the sample; group three has worse but increasing concentrations in TG and the lowest trajectory for HDL-C than the rest and represented 16.8% of the sample.

Group six has the highest trajectory for HDL-C concentrations but optimal in LDL and TG, representing 6.0% of the sample. Group five has optimal values for all considered lipids and represented 13.1% of the sample. Descriptions of each of the multi-trajectory groups are presented in Table 3.

**Table 3:** Description of the multi-trajectory groups of lipid indices

Trajectory group	N (%)	Lipid	Description and mean range from baseline to exam 4^a^
Group 1	3700 (25.1)	LDL-C	Over optimal and slightly decreasing from 120.0 to 112.2 mg/dL
		HDL-C	Low and stable (45.1–44.6 mg/dL)
		Triglycerides	Borderline and slightly increasing from 97.8 to 102.0 mg/dL over time
Group 2	2696 (18.7)	LDL-C	High and decreasing from 172.1 to 152.4 mg/dL over time
		HDL-C	Low and stable (45.0–43.5 mg/dL)
		Triglycerides	Borderline high and increasing from 147.1 to 162.3 mg/dL over time
Group 3	2412 (16.8)	LDL-C	Over optimal and decreasing from 120.2 to 106.0 mg/dL over time
		HDL-C	Very low and stable (37.3–36.8 mg/dL)
		Triglycerides	High and increasing from 200.9 to 219.3 mg/dL over time
Group 4	1355 (9.8)	LDL-C	Over optimal and slightly decreasing from 119.4 to 110.9 mg/dL
		HDL-C	Optimal and stable (59.8–58.7 mg/dL)
		Triglycerides	Borderline high and increasing from 142.6 to 161.0 mg/dL over time
Group 5	1868 (13.1)	LDL-C	Optimal and stable (101.8–98.0 mg/dL)
		HDL-C	High and stable (63.3–63.5 mg/dL)
		Triglycerides	Optimal and stable (69.3–74.0 mg/dL)
Group 6	865 (6.0)	LDL-C	Near-optimal and stable (107.3–103.8 mg/dL)
		HDL-C	Very high and stable (88.9–87.5 mg/dL)
		Triglycerides	Optimal and increasing from 76.8 to 88.4 mg/dL over time
Group 7	1477 (10.5)	LDL-C	High and slightly decreasing from 152.2 to 143.7 mg/dL over time
		HDL-C	High and stable (64.8–63.1 mg/dL)
		Triglycerides	Borderline and increasing from 89.5 to 102.3 mg/dL over time

Triglycerides were analyzed on the log scale, but here they are described in terms of mg/dL

*HDL-C* high-density lipoprotein cholesterol, *LDL-C* low-density lipoprotein cholesterol

^a^Because trajectories are defined in terms of mean lipid levels based on the association between each lipid and age, provided ranges correspond to means at baseline and exam four, and they are not equal to the minimum and maximum lipid values in each group

For descriptive purposes, specific characteristics of each of the seven groups at baseline and follow-up (exam 4) are given in Table 4. There were significant differences (all *p* < 0.05) between all participants' demographic and clinical characteristics in the multi-trajectory groups. Briefly, More than 60 percent of individuals in groups four, five, six, and seven were women, whereas most of the individuals in groups one and three were men. The proportion of current drinking was higher in group 6 that has higher levels of HDL-C. In contrast, the proportions of lipid-lowering medications, hypertension, diabetes mellitus, and BMI were lower in this group than the rest. As expected, individuals in group 5 had the lowest values of TC and LDL-C. Furthermore, the prevalence of family history of CVD at baseline and the incidence rate of CVD at the end of follow-up were lower in this group than the rest.

**Table 4:** Baseline characteristics of participants by multi-trajectory groups of LDL-C, HDL-C, and TG at baseline and exam 4 in the trajectory sample (n = 14,373)

Characteristics	Lipid profile multi-trajectory groups							*p* value
	Group 1	Group 2	Group 3	Group 4	Group 5	Group 6	Group 7	
No. of participants (%)	3700 (25.1)	2696 (18.7)	2412 (16.8)	1355 (9.8)	1868 (13.1)	865 (6.0)	1477 (10.5)	–
Sex, n (%)								
Women	1383 (37.4)	1347 (49.9)	834 (34.6)	1135 (83.8)	1251 (67.0)	755 (87.3)	1134 (76.8)	< 0.001
Men	2317 (62.6)	1349 (50.1)	1578 (65.4)	220 (16.2)	619 (33.0)	110 (12.7)	343 (23.2)	
Race, n (%)								
White/Caucasian	2271 (61.4)	1997 (74.1)	1716 (71.1)	972 (71.7)	1094 (58.6)	591 (68.3)	952 (64.5)	< 0.001
African American	962 (26)	512 (19)	260 (10.8)	175 (12.9)	583 (31.2)	227 (16.3)	446 (30.2)	
Hispanic/Latino	298 (8.1)	137 (5.1)	290 (12.0)	130 (9.6)	114 (6.1)	30 (3.5)	45 (3.1)	
Asian/Chinese	169 (4.6)	50 (1.9)	146 (6.1)	78 (5.8)	77 (4.1)	17 (2.0)	34 (2.3)	
Baseline								
Age, y	56.3 (8.6)	56.3 (6.9)	55.6 (7.9)	56.7 (7.9)	56.5 (9.2)	56.6 (8.5)	56.1 (7.5)	0.005
BMI, kg/m^2^	28.1 (5.1)	28.2 (4.7)	29.3 (4.8)	27.2 (5.3)	26.2 (5.4)	24.4 (4.4)	26.6 (5.1)	< 0.001
TC, mg/dL	184.7 (27.9)	246.5 (34.6)	197.7 (32.6)	207.7 (28.6)	179.0 (26.3)	211.5 (32.6)	234.8 (31.4)	< 0.001
LDL-C, mg/dL	120.0 (25.7)	172.1 (31.5)	120.2 (29.1)	119.4 (26.2)	101.8 (24.2)	107.3 (30.1)	152.2 (29.1)	< 0.001
HDL-C, mg/dL	45.1 (7. 6)	45.0 (8.6)	37.3 (7.6)	59.8 (9.4)	63.3 (9.7)	88.9 (14.5)	64.8 (10.2)	< 0.001
TG, mg/dL	97.8 (33.7)	147.1 (54.1)	200.9 (68.9)	142.6 (51.3)	69.3 (21.8)	76.8 (31.0)	89.5 (29.2)	< 0.001
Lipid-lowering drug	608 (16.6)	656 (24.5)	479 (20.0)	244 (18.1)	238 (12.8)	103 (12.0)	281 (19.1)	< 0.001
SBP, mmHg	121.3 (19.0)	122.1 (17.8)	122.2 (17.0)	121.5 (18.8)	118.0 (19.3)	118.0 (19.1)	120.8 (18.9)	< 0.001
DBP, mmHg	73.1 (10.4)	73.4 (10.4)	73.5 (9.9)	71.3 (9.9)	70.7 (10.4)	70.2 (10.7)	72.6 (10.6)	< 0.001
Hypertension	1207 (32.7)	913 (33.9)	852 (35.4)	430 (31.8)	506 (27.2)	209 (24.2)	468 (31.8)	< 0.001
Antihypertensive drug	1008 (27.3)	750 (27.8)	721 (29.9)	347 (25.6)	442 (23.7)	172 (19.9)	370 (25.1)	< 0.001
Diabetes mellitus,	345 (9.3)	247 (9.2)	366 (15.2)	85 (6.3)	112 (6.0)	30 (3.5)	84 (5.7)	< 0.001
Current smoking	687 (18.6)	580 (21.6)	501 (20.8)	221 (16.3)	293 (15.7)	132 (15.3)	220 (14.9)	< 0.001
Current drinking	2118 (57.6)	1540 (57.3)	1416 (58.8)	804 (59.6)	1141 (61.3)	605 (70.0)	886 (60.4)	< 0.001
Physical activity (MET-minutes/week)	1070.1 (1715.2)	801 (1432.4)	913.7 (1399.0)	966.5 (1645.4)	1226.3 (1786.3)	1065.2 (1568.2)	874.4 (1280.8)	< 0.001
Calorie intake (Kcal)	1652.2 (745.7)	1631.7 (703.2)	1697.5 (755.1)	1489.0 (638.0)	1520.0 (673.2)	1485.9 (629.1)	1529.5 (695.1)	< 0.001
CVD family history	2024 (58.8)	1641 (66.3)	1389 (62.1)	817 (63.7)	1000 (58.1)	474 (59.5)	834 (61.5)	< 0.001
Follow-up (exam 4)								
Age, y	63.5 (7.9)	64.5 (6.4)	63.0 (7.3)	64.0 (7.2)	63.6 (8.4)	64.2 (7.6)	64.0 (7.0)	< 0.001
BMI, kg/m^2^	28.9 (5.5)	29.3 (5.0)	30.2 (5.0)	28.3 (5.7)	26.9 (5.7)	25.2 (4.9)	27.6 (5.4)	< 0.001
TC, mg/dL	176.9 (28.8)	224.9 (35.9)	185.1 (34.1)	201.6 (30.1)	176.4 (25.4)	208.6 (30.6)	226.5 (28.4)	< 0.001
LDL-C, mg/dL	112.2 (25.8)	152.4 (31.4)	106.0 (27.4)	110.9 (26.5)	98.0 (23.4)	103.8 (28.0)	143.7 (25.6)	< 0.001
HDL-C, mg/dL	44.6 (7.9)	43.5 (8.2)	36.8 (7.6)	58.7 (10.1)	63.5 (9.9)	87.5 (15.0)	63.1 (10.1)	< 0.001
TG, mg/dL	102.0 (35.2)	162.3 (66.9)	219.3 (103)	161.0 (63.0)	74.0 (24.9)	87.5 (40.3)	102.3 (36.4)	< 0.001
Lipid-lowering drug	1178 (32.4)	1291 (48.2)	937 (39.4)	468 (35.0)	420 (22.9)	211 (24.7)	505 (34.6)	< 0.001
SBP, mmHg	124.4 (18.7)	128.4 (19.3)	125.8 (18.2)	127.1 (20.1)	122.0 (19.9)	123.7 (20.0)	126.6 (20.2)	< 0.001
DBP, mmHg	71.0 (9.8)	70.8 (10.3)	70.6 (10.1)	69.7 (10.2)	69.6 (10.4)	69.1 (9.8)	70.8 (10.4)	0.045
Hypertension	1677 (45.8)	1357 (50.7)	1188 (49.7)	658 (48.9)	746 (40.3)	331 (38.5)	684 (46.6)	< 0.001
Antihypertensive drug	1570 (43.1)	1259 (46.9)	1114 (46.8)	553 (41.3)	657 (35.8)	286 (33.5)	583 (39.8)	< 0.001
Diabetes mellitus	587 (16.1)	471 (17.6)	681 (28.4)	152 (11.3)	177 (9.6)	43 (5.0)	115 (7.9)	< 0.001
Current smoking	502 (13.7)	374 (13.9)	327 (13.6)	156 (11.5)	219 (11.8)	94 (10.9)	151 (10.3)	< 0.001
Current drinking	1737 (42.3)	1239 (46.3)	1118 (46.7)	668 (49.6)	993 (53.5)	553 (62.1)	759 (51.9)	< 0.001
CVD incidence rate^a^	7.50	13.12	11.20	6.23	4.33	5.70	7.35	

Numbers represent mean (standard deviation) for continuous and frequency (%) for categorical variables

ANOVA or Kruskal–Wallis for continuous variables and chi-square for categorical variables

*BMI* body mass index, *TC* total cholesterol, *LDL-C* low-density lipoprotein cholesterol, *HDL-C* high-density lipoprotein cholesterol, *TG* triglycerides, *SBP* systolic blood pressure, *DBP* diastolic blood pressure, *CVD* cardiovascular disease

^a^Per 1000 person-years; starting to follow up at examination cycle 4 through December 2014

### Multi-trajectory groups of lipid indices and incident cardiovascular disease, heart failure, and all-cause mortality

During the follow-up after examination cycle 4 (median = 13.5 years), there were 1133 incident CVD events, 1075 heart failure, and 2315 deaths. Table 4 presents hazard ratios (HRs) and 95% confidence intervals (CIs) of multi-trajectory groups on incident CVD, heart failure, and all-cause mortality. As expected, groups with worse trajectories for the considered lipid indices had a higher risk of developing CVD (Table 5). The proportion of incident events was higher among individuals whose LDL-C levels are the highest and decreasing, TG levels are borderline high and increasing, and HDL-C levels are low (Fig. 2, group 2). Compared with the individuals with an optimal and stable level of the considered lipid indices (Fig. 2, group 5), those in the other multi-trajectory groups were at a statistically significant increased risk of incident CVD after adjusting for sex, age, race, lipid-lowering medication use, diabetes mellitus, hypertension, body mass index, smoking status, and alcohol consumption (Table 5). However, this risk was not similar across multi-trajectory groups. Individuals with the highest but decreasing LDL-C and borderline high and rising TG levels over time (Fig. 2, group 2) were at the highest risk than those in other groups with a 2.22-fold risk of CVD. Also, those with the highest and increased triglyceride levels over time, over optimal and decreasing LDL-C levels, and the lowest HDL-C profile (Fig. 2, group 3) had a nearly 1.84 times CVD risk. Furthermore, individuals with the highest HDL-C level (Fig. 1, group 6) had a 1.45-fold risk of CVD compared to those with the optimal levels of lipid indices (Fig. 2, group 5). Furthermore, only the highest HDL-C level (Fig. 2, group 6) was significantly associated with incident heart failure with a 1.5 times increased risk (95% CI 1.07–2.06).

**Table 5:** Associations between multi-trajectory groups of lipid indices and incident CVD, heart failure, and all-cause mortality

Trajectory group	Incident CVD			Incident heart failure			All-cause mortality		
	Cases/n (%)	Hazard ratio (95% CI)	*p* value	Cases/n (%)	Hazard ratio (95% CI)	*p* value	Cases/n (%)	Hazard ratio (95% CI)	*p* value
Group 1	239/3131 (7.6)	1.40 (1.08–1.82)	0.012	242/3099 (7.8)	1.16 (0.91–1.49)	0.230	521/3131 (16.6)	0.90 (0.77–1.05)	0.202
Group 2	344/2134 (16.1)	2.22 (1.72–2.87)	< 0.001	270/2108 (12.8)	1.21 (0.95–1.55)	0.121	595/2134 (27.9)	1.02 (0.87–1.18)	0.819
Group 3	226/1991 (11.4)	1.84 (1.40–2.41)	< 0.001	197/1962 (10.0)	1.26 (0.97–1.63)	0.087	387/1991 (19.4)	0.95 (0.87–1.19)	0.634
Group 4	81/1179 (6.9)	1.39 (1.01–1.87)	0.041	92/1169 (7.9)	1.17 (0.87–1.57)	0.296	179/1179 (15.2)	0.96 (0.81–1.14)	0.393
Group 5	75/1653 (4.5)	1.00 (Reference)	–	90/1637 (5.4)	1.00 (Reference)	–	247/1653 (14.9)	1.00 (Reference)	–
Group 6	51/765 (6.7)	1.45 (1.02–2.08)	0.040	61/755 (8.1)	1.50 (1.07–2.06)	0.018	145/765 (19.0)	1.21 (0.99–1.49)	0.068
Group 7	117/1276 (9.2)	1.56 (1.17–2.09)	0.003	123/1259 (9.8)	1.23 (0.94–1.62)	0.135	241/1276 (18.9)	0.90 (0.75–1.07)	0.228

All the models were adjusted for age, sex, race, lipid-lowering medication use, diabetes mellitus, hypertension, body mass index, smoking status, and alcohol consumption at exam 4. The interaction of sex and trajectory groups was significant just for groups 1, 2, and 3 (p < 0.05) and not significant for others

*CI* confidence interval, *CVD* cardiovascular disease

We did not observe a statistically significant difference in the risk of death between individuals in the referent group and those in other multi-trajectory groups (Table 5). Nevertheless, the multi-trajectory group with the highest HDL-C level increased the risk of death by 20 percent with a marginal significance.

Results from sensitivity analyses additionally adjusted for total caloric intake and physical activity at the baseline exam produced similar findings as our primary analyses, except for multi-trajectory groups 2 and 3 that demonstrated to be significantly associated with incident heart failure with 1.31 and 1.42 times increased risk (95% CI 1.03–1.68 and 1.09–1.84), respectively.

## Discussion

This large, pooled cohort study investigated heterogeneity in lipid profile multi-trajectories among individuals aged 45 to 84 years. Using group-based multi-trajectory analyses of the longitudinal data across the four examination cycles, we identified seven distinct multi-trajectory groups of LDL-C, HDL-C, and TG concentrations. Approximately 80% of participants exhibited decreasing LDL-C levels but rising TG levels and relatively stable levels of HDL-C.

We revealed that compared with the individuals presenting with optimal and stable levels of these lipids across the life course, those in the other multi-trajectory groups were at an increased risk of incident CVD after adjusting for confounders. However, individuals in different groups showed a different risk of CVD. We also observed that individuals presenting with very high HDL-C levels throughout the adult life course were at a significantly increased risk of incident CVD and heart failure, and a marginally significant risk of death than those with optimal lipid profile, after adjusting for confounders. Besides, In contrast with very high levels of HDL-C, the risk of death and incidence of heart failure did not differ across other multi-trajectories compared to the optimal lipid indices levels.

Although numerous studies have been suggested the association of lipids with the risk of CVD, the trajectories we identified in this study provide new insights for the common progression of lipid indices that could be expected to be observed during the age of 45 to 84 years in relatively healthy adults. We used a person-centered, multi-trajectory approach that modeled the common progressions of LDL-C, HDL-C, and TG levels. This model is considerably different because for identifying and monitoring the various lipid indices progressions herein, the usage of lipid-lowering medications simultaneously incorporated and accounted for correlation of lipids within the same participant and over time. Such an approach defines a trajectory group in terms of trajectories for multiple indicators, not just one indicator. In so doing, the model efficiently represents the interrelationship of numerous clinically relevant indicators. Such approaches visually represent distinct groups of individuals who display unique lipid indices patterns over time [20].

Previous studies using trajectory modeling only investigated the development of each lipid component separately [3, 10–12, 23]. To date, only one previous study by Dayimu et al. has considered the trajectory of these lipids jointly. It has shown three distinct trajectory classes (U-shape class, progressing, and inverse U-shape) in a Chinese population aged 20 to 60 years [9]. Our findings widen previous evidence in this field by showing that lipids can be jointly categorized into seven different multi-trajectory groups over the age of 45–84.

Interestingly, we found a decreasing trend in LDL-C over time, although only 40 percent of participants were on lipid-lowering agents. Moreover, when we excluded those, who had been receiving lipid-lowering medications from our analysis, the trend remained relatively unchanged (Additional file 2: Fig. S1). Thus, this favorite trend might also be attributable to the FDA’s regulations and national guidelines regarding Americans’ dietary fat and cholesterol intake [24–26]. Yet, even after falls in LDL-C, a considerable amount of CVD risk remains. So, these findings suggest the impact of the increasing trend of TG on residual cardiovascular risk [27, 28].

Although the exact role of TG in inducing ASCVD has been controversial, the evidence supporting the association of elevated concentrations of triglyceride-rich lipoproteins or remnant cholesterol, reliably marked by raised triglycerides, and cardiovascular disease and all-cause mortality, is increasing. Taken together, numerous observational and genetic studies strongly support the association of mild-to-moderately raised triglyceride concentrations and CVD risk [29–33]. Previous studies have confirmed that elevated TG levels even below 150 mg/dL, previously considered “optimal” levels, were associated with increased CVD risk [34–36]. This evidence suggesting that a biologically “optimal” level may be even lower for TG as American Heart Association also indicated that an “optimal” fasting TG level is less than 100 mg/dL [37].

We also revealed that individuals with a higher TG level had a lower HDL-C, depicted in the trajectory plot (Fig. 2, groups 2 and 3), and were at a greater risk of incident CVD than other individuals. Our finding is consistent with evidence from some genetic studies and randomized trials suggesting that low HDL cholesterol might merely be a long-term marker of raised triglycerides and remnant cholesterol, not a cause of CVD [38–42]. A Mendelian randomization study using genetic variants that affect the remnant cholesterol concentrations, HDL cholesterol, or both demonstrated that a 1 mmol/L (39 mg/dL) increase in remnant cholesterol is associated with a 2.8-fold risk for ischemic heart disease, independent of reduced HDL cholesterol [31]. Another study also showed that increasing TGs were associated with more significant increases in CVD risk among individuals with higher HDL-C levels [34].

Our findings support the associations of elevated TG with the risk of future CVD, and the use of trajectory of TGs to identify high-risk individuals for CVD events that are in accordance with some studies showing that using several TG measurements improved prediction of CVD risk more than a single TG measurement [34]. However, whether TGs are an independent cause for incident CVD or serve as a marker for other risk factors remains unclear. Evidence from some genetic studies suggested that the risk associated with elevated TGs might be because of their association with elevations in non–HDL-C or apolipoprotein B (apo B)-containing lipoproteins [43].

Finally, some randomized trials also showed an increased risk for CVD in statin-treated patients with elevated TGs [44–47]. A randomized trial known as Reduction of Cardiovascular Events with EPA-Intervention Trial (REDUCE-IT) assessed the impact of TG-lowering using Icosapent ethyl among statin-treated patients with TGs ≥ 135 and < 500 mg/dL, and with a history of CVD, diabetes, or other risk factors. Its finding showed that lowering TG was significantly associated with a lower risk of CVD [48]. Recently, a systematic review and meta-regression analysis of randomized controlled trials also showed a significant association between TG-lowering and CV risk reduction, even after adjusting for LDL-C lowering, although the effect attenuated when REDUCE-IT was excluded from the analysis [49].

Another contribution of this study is that we found a group of individuals (approximately 6%) with very high and stable levels of HDL-C but optimal LDL-C and TG levels that were at risk of CVD, heart failure, and even a marginally significant risk of death. Our finding is consistent with evidence from clinical studies showing that both serum HDL-C concentration/quantity and its qualities/properties can play a critical role in determining its overall effects and, hence, its association with clinical outcomes [50–52]. So, not only HDL serum cholesterol concentrations but also a range of other properties, including its particle size and composition, its Apo lipoprotein content, its enrichment with proinflammatory properties, and its functional capacity, can play a critical role in determining its overall effects and hence its association with clinical outcomes [50, 53].

Finally, compared to previous studies that reported associations between lipid profile trajectories and all-cause mortality [9, 10], we did not observe a statistically significant association of multi-trajectory groups with death. However, the highest HDL-C but optimal LDL-C and TG profile had a marginally significant risk of death. Several large-scale prospective cohort studies have recently revealed a U-shaped association between HDL-C levels and all-cause mortality, indicating that both very high and low levels are associated with an increased risk of death [54, 55]. Some other studies also reported that high LDL-C and HDL-C levels are inversely associated with mortality, especially in older people [56, 57].

This study has several strengths, including a pooled data set from two large representative cohort studies with multiple examinations across adulthood. Besides, continuous follow-up for collecting data on various variables and CVD and death events at ARIC and MESA studies allowed us for inclusive adjustment for risk factors. A key strength of this study is the use of an innovative multi-trajectory modeling technique to identify subgroups of longitudinal lipid profile trajectories based on multiple lipids. The multi-trajectory analysis used in this study incorporates the inter-correlations among the multiple lipids to improve the accuracy of individual-specific probabilities of group membership, while the conventional group-based trajectory analysis clusters longitudinal trajectories based on one outcome. Despite these strengths, this study has limitations. First, we used repeated measurements of lipids just over the four examination cycles; we could not use the examination cycle 5 to ensure sufficient follow-up time for the prospective analyses, so longer variation in the lipid pattern might be missed. However, some evidence clarifies that at least three unique time points are required for the trajectory approach [58, 59]. Second, despite the large sample size included in this study, the number of individuals with extremely high HDL-C concentrations was relatively small (~ 6% of the total sample), especially in the stratified analyses, limiting statistical power. Third, in common with all modeling approaches, there are limitations attendant to trajectory models. Although several model diagnostic criteria were proposed as guides, the problem of specifying the correct number of groups has not been entirely resolved. Fourth, it should also be noted that GBTM attempts to provide potentially clinically meaningful trajectory groups based on the available data, and they should not be taken to exist literally.

In conclusion, the present observations provide a comprehensive depiction of the joint progression of lipid indices over time. The trajectories and risk of CVD identified in this study demonstrated that despite a decline in LDL-C over time, a significant amount of residual risk for CVD remains. These findings suggest the impact of the increasing trend of TG on CVD risk and emphasize the importance of assessing the lipid levels at each visit and undertaking potential interventions that lower triglyceride concentrations to reduce the residual risk of CVD. Since our findings are exploratory and do not address treatment questions, future research should focus more on the effects of TG-lowering strategies in reducing residual cardiovascular risk, even among those with the optimal LDL-C level.

## Supplementary Information

**Additional file 1: Table S1.** Correlation coefficients among lipid profile and lifestyle factors.

**Additional file 2: Figure S1.** Multi-trajectory groups of LDL-C, HDL-C, and TG among participants not on lipid-lowering treatment. Dots show group-specific mean observed levels while solid lines represent fitted trajectories. Lipids were modeled as a function of age.

## Data Availability

The datasets used during the current study are available from the corresponding author on reasonable request.

## Abbreviations

CVD: Cardiovascular disease
LDL-C: Low-density lipoprotein cholesterol
HDL-C: High-density lipoprotein cholesterol
TG: Triglyceride
BMI: Body mass index
MI: Myocardial infraction
CHD: Chronic heart disease
ARIC: Atherosclerosis risk in communities
MESA: Multi-ethnic study of atherosclerosis
GBTM: Group-based trajectory model
AvePPj: Average posterior probability of assignment for each group j
OCCj: Odds of correct classification
FDA: Food and Drug Administration
