# Supplementary material for: Multi-trajectories of lipid indices with incident cardiovascular disease, heart failure, and all-cause mortality: 23 years follow-up of two US cohort studies
Source: J Transl Med. 2021 Jul 3;19:286. doi: 10.1186/s12967-021-02966-4 (PMC8254336; doi:10.1186/s12967-021-02966-4)
Supplement: Supplementary file 1 — Additional file 1: Table S1. Correlation coefficients among lipid profile and lifestyle factors. [file 12967_2021_2966_MOESM1_ESM.docx]

| **Table S1. Correlation coefficients among lipid profile and lifestyle factors.** | | | | | | | | |
| --- | --- | --- | --- | --- | --- | --- | --- | --- |
| Variable | LDL-C | HDL-C | TG | physical activity | total caloric intake | Smoking status | Alcohol consumption | VIF |
| LDL-C | 1.0000 |  |  |  |  |  |  | 1.04 |
| HDL-C | -0.1489* | 1.0000 |  |  |  |  |  | 1.26 |
| TG | 0.1465* | -0.4320* | 1.0000 |  |  |  |  | 1.24 |
| physical activity | -0.0709* | 0.0050 | -0.0361* | 1.0000 |  |  |  | 1.01 |
| total caloric intake | 0.0165 | -0.1019* | 0.0567* | -0.0065 | 1.0000 |  |  | 1.02 |
| Smoking status | 0.0314* | -0.0967* | 0.0501 | -0.0239* | 0.0761* | 1.0000 |  | 1.08 |
| Alcohol consumption | -0.0283* | 0.0151 | 0.0060 | 0.0772* | 0.0731* | 0.2465* | 1.0000 | 1.08 |
| Mean VIF |  |  |  |  |  |  |  | 1.11 |

LDL-C, low-density lipoprotein cholesterol; HDL-C, high-density lipoprotein cholesterol; TG, triglycerides; VIF, variance inflation factor.

* P<0.05
