# Supplementary material for: Multi-trajectories of lipid indices with incident cardiovascular disease, heart failure, and all-cause mortality: 23 years follow-up of two US cohort studies
Source: J Transl Med. 2021 Jul 3;19:286. doi: 10.1186/s12967-021-02966-4 (PMC8254336; doi:10.1186/s12967-021-02966-4)
Supplement: Supplementary file 2 — Additional file 2: Figure S1. Multi-trajectory groups of LDL-C, HDL-C, and TG among participants not on lipid-lowering treatment. Dots show group-specific mean observed levels while solid lines represent fitted trajectories. Lipids were modeled as a function of age. [file 12967_2021_2966_MOESM2_ESM.docx]

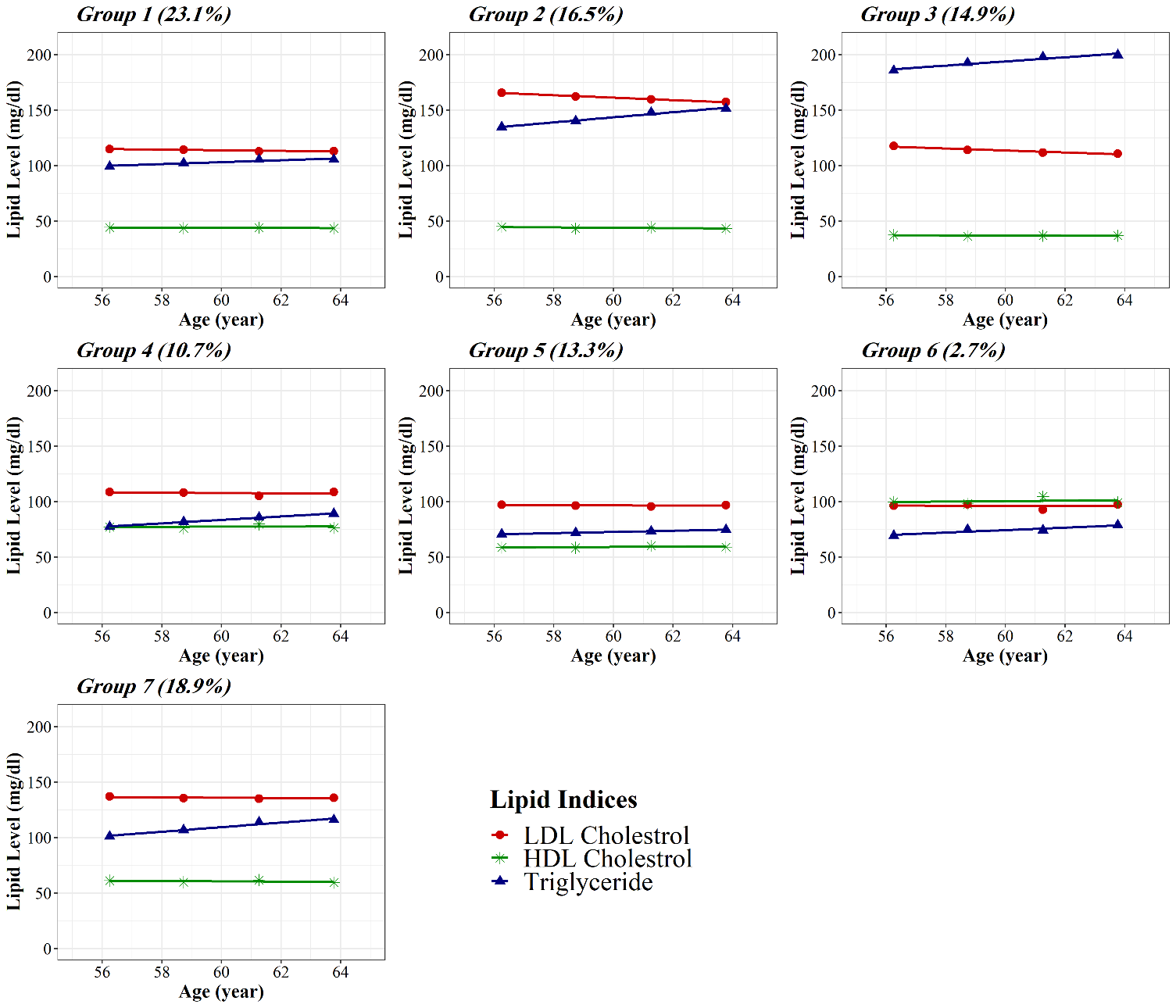


**Figure S1.** Multi-trajectory groups of LDL-C, HDL-C, and TG among participants not on lipid-lowering treatment. Dots show group-specific mean observed levels while solid lines represent fitted trajectories. Lipids were modeled as a function of age.
